# Amyotrophic Lateral Sclerosis: Molecular Mechanisms, Biomarkers, and Therapeutic Strategies

**DOI:** 10.3390/antiox10071012

**Published:** 2021-06-24

**Authors:** Xiaoming Yang, Yanan Ji, Wei Wang, Lilei Zhang, Zehao Chen, Miaomei Yu, Yuntian Shen, Fei Ding, Xiaosong Gu, Hualin Sun

**Affiliations:** 1 Key Laboratory of Neuroregeneration of Jiangsu and Ministry of Education, NMPA Key Laboratory for Research and Evaluation of Tissue Engineering Technology Products, Jiangsu Clinical Medicine Center of Tissue Engineering and Nerve Injury Repair, Co-Innovation Center of Neuroregeneration, Nantong University, Nantong 226001, China; sammy@ntu.edu.cn (X.Y.); 2022310030@stmail.ntu.edu.cn (Y.J.); ntwangwei911@ntu.edu.cn (W.W.); 1924310007@stmail.ntu.edu.cn (L.Z.); 2022310042@stmail.ntu.edu.cn (Z.C.); 1931510003@stmail.ntu.edu.cn (M.Y.); syt517@ntu.edu.cn (Y.S.); 2 School of Biology and Basic Medical Sciences, Medical College of Soochow University, Suzhou 215006, China

**Keywords:** ALS, pathogenesis, biomarkers, treatment strategies

## Abstract

Amyotrophic lateral sclerosis (ALS) is a neurodegenerative disease with the progressive loss of motor neurons, leading to a fatal paralysis. According to whether there is a family history of ALS, ALS can be roughly divided into two types: familial and sporadic. Despite decades of research, the pathogenesis of ALS is still unelucidated. To this end, we review the recent progress of ALS pathogenesis, biomarkers, and treatment strategies, mainly discuss the roles of immune disorders, redox imbalance, autophagy dysfunction, and disordered iron homeostasis in the pathogenesis of ALS, and introduce the effects of RNA binding proteins, ALS-related genes, and non-coding RNA as biomarkers on ALS. In addition, we also mention other ALS biomarkers such as serum uric acid (UA), cardiolipin (CL), chitotriosidase (CHIT1), and neurofilament light chain (NFL). Finally, we discuss the drug therapy, gene therapy, immunotherapy, and stem cell-exosomal therapy for ALS, attempting to find new therapeutic targets and strategies. A challenge is to study the various mechanisms of ALS as a syndrome. Biomarkers that have been widely explored are indispensable for the diagnosis, treatment, and prevention of ALS. Moreover, the development of new genes and targets is an urgent task in this field.

## 1. Background

Amyotrophic lateral sclerosis (ALS) is a fatal neurodegenerative disease in adults that was first described by a neurobiologist Jean-Martin Charcot in the 1870s and was originally called Charcot’s triad. This disease is also called Lou Gehrig’s disease in the United States, to commemorate the baseball players who were diagnosed with it in the 1930s [1]. ALS mainly involves upper motor neurons (UMN) and lower motor neurons (LMN), manifested as a progressive paralysis. Nervous system symptoms include spasm, muscle stiffness, hyperreflexia, and pathological reflexes, which are signs of UMN involvement; muscle weakness and atrophy are signs of LMN injury [2]. ALS usually progresses rapidly, and a death of respiratory failure occurs within one to five years after the onset of the disease. Immunohistochemical detection indicates the accumulation of ubiquitinated proteins in ALS motor neurons and glial cells [3]. Most cases of ALS are sporadic (sALS), and 10% of ALS is familial (fALS) [4]. In the past 20 years, increasing evidence has shown that patients initially diagnosed with ALS present with clinical symptoms of frontotemporal dementia (FTD), indicating that the two diseases share common symptoms and biological characteristics [5]. Extending the life span of ALS patients can deepen the understanding of its pathogenesis, promoting the development of early and specific diagnostic methods. There is an urgent need to develop a treatment plan, not only to slow the progression of the disease, but also to cope with the secondary consequences of malnutrition and respiratory failure [6]. Up to now, scientists have conducted a lot of related studies, but unfortunately the pathogenesis of ALS is still unclear.

Through this review, we can learn about the research progress in ALS pathogenesis, biomarkers and treatment strategies in recent years, and have a systematic understanding of ALS. The latest discovery of the underlying mechanism of ALS will help reduce the incidence of ALS, slow down the progression of the disease, and find new targets for the treatment of ALS.

## 2. Pathogenesis of ALS

**Figure 1:**
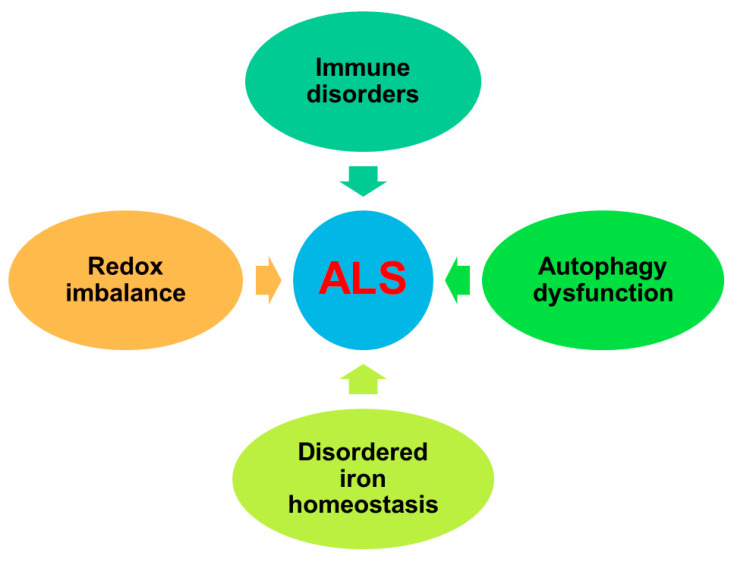
Pattern diagram of pathogenic factors in ALS.

In the past few decades, a breakthrough has been made in understanding the pathogenesis of ALS. Immune disorders, redox imbalance, autophagy dysfunction, and disordered iron homeostasis are important factors involved in the pathogenesis of ALS (Figure 1).

### 2.1. Immune Disorders

The immune system is an important part involved in the pathogenesis of ALS. Studies have shown that in sALS patients, there are immunological changes in the blood and changes in immune cell levels [7] and increased levels of circulating monocytes and macrophages. Moreover, the degree of activation is directly related to the disease progression [8]. In ALS, a dynamic interaction exists between innate immune cells (i.e., microglia and astrocytes), which may cause progressive damage to motor neurons. Innate and adaptive immune responses are related to progressive neurodegeneration. However, although T-cells appear to harmful effects at the early stages of ALS, immune activation pathways are beneficial to promote the repair of damaged nerve tissues [9]. Activated microglia can be found in the motor cortex and spinal cord of ALS patients, and the activation intensity is related to the severity of UMN damage and T cell infiltration [10,11]. These activated microglia can highly express complement receptors and MHC molecules in the central nervous system [12]. Compared with healthy controls, there are changes in systemic inflammation markers and immune cell populations of ALS patients. The levels of neutrophils, CD4 and CD8 lymphocytes also vary in ALS patients [13,14,15]. The expansion of a hexanucleotide repeat G4C2 motif in the chromosome 9 open reading frame 72 (C9orf72) gene is the most common cause of ALS [16]. An increase in immunoreactive microglia and peripheral inflammation can be observed in C9orf72-ALS patients. There is an interaction between the intracellular pathway induced by C9orf72 expansion and the innate immune inflammasome complex. The innate immune inflammasome complex is an intracellular receptor that can induce inflammation in the cellular stress response [17]. In a word, the pathogenesis of ALS is intricately related to immune cell population, complements, cytokines, and a series of immune inflammatory markers. We can ascertain that the disorder of the immune system is an important cause of ALS; however, the specific molecular mechanisms need to be further explored.

### 2.2. Redox Imbalance

Increasing evidence highlights the importance of redox imbalance in the pathogenesis of ALS. For example, a mutant superoxide dismutase 1 (SOD1) disrupts the redox homeostasis of ALS through the abnormal production of reactive oxygen species (ROS) and reactive nitrogen (RNS) and the formation of misfolded protein aggregates [18,19]. Human apurinic/apyrimidinic endonuclease 1 (APE1), a redox-regulation protein for transcription factors, is upregulated in motor neurons and astrocytes from spinal cord of ALS patients [20]. The mutant SOD1^G93A^ confines the localization of APE1 and inhibits redox homeostasis [21]. Thioredoxin-related transmembrane protein 2 (TMX2) is an important sensor to maintain redox homeostasis in cells. Deletion of TMX2, a protective modifier for the toxicity of C9orf72 DPR (dipeptide repeat), can suppress PR_20_-induced cytotoxicity and synthetic polymers of Proline-Arginine [22]. Mutations in protein disulfide isomerases (PDIs) are also associated with the risk of ALS [23]. Owing to the redox activity PDIs play a protective role in cell and zebrafish models [24]. Oxidative stress (OS) plays an important role in the occurrence and development of ALS and is also closely related to the degeneration of motor neurons in ALS. ALS can increase the levels of oxidative stress biomarkers in the cerebrospinal fluid (CSF), plasma, and urine. Many environmental risk factors can lead to systemic oxidative stress, and increased systemic oxidative stress may accelerate the progression of ALS disease [25]. SOD1 can lead to an increase in the level of oxidative stress through a loss of function mechanism. The remaining wild-type SOD1 may become the target of oxidative modification, which then dissociates from dimers into monomers, and further forms aggregates with the toxicity of the SOD1 mutation [26,27]. Oxidative stress-mediated protein damage, lipid peroxidation, and DNA and RNA oxidation can be observed in ALS patients, as well as oxidative stress biomarkers [28]. In addition, mitochondrial dysfunction is directly related to oxidative stress, which contributes to the increase of ROS/RNS. ROS can cause mitochondrial DNA mutations, membrane permeability, and calcium homeostasis, and enhance lipid oxidation and protein carbonylation, leading to various neurodegenerative diseases including ALS [29,30]. Overall, redox imbalance is a well-recognized phenomenon in the pathogenesis of ALS. Many studies in vivo and in vitro have proved that redox imbalance is one of the important factors of nervous system degeneration. Some unknown antioxidants are expected to slow the progression of the disease or help patients extend their lives.

### 2.3. Autophagy Dysfunction

Proteasomal degradation cannot proceed during protein folding, so aggregated proteins are poor substrates for the proteasome. This type of proteins is produced through autophagy transformation. Autophagy is therefore considered to be an important neuroprotective pathway. However, patients with ALS appear to have specific autophagy dysfunction in glial cells [31]. Many ALS-related genes are found to be related to autophagy. For example, exome sequencing has indicated that a mutation in valosin-containing protein (VCP) is the cause of fALS [32], and VCP is related to autophagy of stress particles. Stress particles are involved in the translation and decay of mRNA and also related to ribonucleoprotein (RNP) particles in embryos, neurons, and inclusion bodies of some degenerative diseases [33]. VCP can also maintain the stability of lysosomes [34]. The protein product of C9orf72 gene participates in the regulation of unc-51-like kinase 1 (ULK1), leading to the initiation of autophagy accordingly [35]. C9orf72 binds Smith–Magenis syndrome chromosome region, candidate 8 (SMCR8), locates in the lysosome, regulates the mechanistic target of rapamycin complex 1 (mTORC1) signal transduction, and plays a role in the lysosome [36]. Autophagy regulators TFEB (a coordinator of autophagy induction and lysosomal biogenesis) [37] and Beclin1 (an autophagy initiator involved in autophagosome production) continue to be upregulated in the spinal cord of SOD1^G93A^ mice [38]. TANK-binding kinase 1 (TBK1) is a polykinase that regulates inflammation and autophagy [39]. As previously reported, TBK1 is colocalized with optineurin on protein aggregates in HeLa cells in vitro and in the SOD1 transgenic mouse model of ALS [40]. Kinesin family member 5A (KIF5A) acts as a key target for trimethyltin chloride (TMT) to damage autophagic flux. TMT can reduce the expression of KIF5A protein, destroy the interaction between KIF5A and lysosome, and disturbs the axon transport of lysosome. In addition, overexpression of KIF5A restores the axon transport, increases lysosomal dysfunction, and antagonizes TMT-induced neurotoxicity in vitro [41]. Cyclin F gene (CCNF) encodes cyclin F, and its pathogenic mutation destroys Lys48-specific ubiquitination, leading to substrate accumulation and deficiency in the autophagy mechanism. A single missense mutation in cyclin F can cause protein hyperubiquitination, indirectly impairing the autophagy degradation pathway, which is related to the pathogenesis of ALS [42]. In recent years, when researchers study the pathogenesis of ALS, they mostly focus on neurons, but ignore the potential effect of autophagy on the nervous system. This may allow us to open up a new treatment against autophagy for ALS.

### 2.4. Disordered Iron Homeostasis

Iron is essential for all mammalian cells, but it is very toxic. The mouse model of ALS has been used to confirm the role of iron in the pathogenesis of ALS. One of ALS characteristics is that motor neuron dysfunction in the spinal cord can impact cellular iron homeostasis, forming a vicious circle and exacerbating oxidative damage [43]. Increasing evidences have shown that the accumulation of iron is related to the occurrence and development of ALS, and disordered iron homeostasis plays an important role in the pathogenesis of ALS [44]. Studies have shown that disordered iron homeostasis in the central nervous system contributes to disease progression in the mouse ALS model [45]. In addition to motor dysfunction, ALS patients also have sensory dysfunction in the early stage of the disease [46]. Disorders of metal homeostasis, including iron, may promote mitochondrial dysfunction of astrocytes in sensory ganglia, causing sensory neuron disorders and sensory symptoms. This suggests that metal homeostasis disorders and mitochondrial dysfunction may be the mechanisms underlying the pathological changes related to ALS exercise and extra-exercise symptoms [47]. Administration of Jaeumganghwa-Tang (JGT) has been reported to significantly delay motor function damage and reduce oxidative stress in hSOD1^G93A^ transgenic mice (human mutant SOD1 transgenic mice). JGT can down-regulate Toll-like receptor 4 (TLR4) related signal proteins and improve the iron homeostasis in the spinal cord of hSOD1^G93A^ mice to effectively improve the neuroinflammatory mechanism [48]. In addition, the imbalance of ROS production in ALS patients, which may be directly caused by the mutant SOD1 or indirectly caused by other mechanisms, maybe triggers the damage to the Fe-S clusters, or leads to the mechanism of iron regulatory protein-iron responsive element (IRE-IRP) and inactivation of mitochondrial enzymes [49]. Overall, iron is an essential element for many functions of the brain, which plays a role in neuroprotection and repair in neurodegenerative diseases. Various iron chelators that have been explored may be a promising therapeutic method.

## 3. Biomarkers of ALS

The markers of ALS are very important for the prediction of disease development, the judgment of prognosis, and the choice of treatment strategies. The market of ALS mainly includes RNA binding proteins (RBPs), ALS-related genes, and non-coding RNA (Table 1).

### 3.1. RNA Binding Proteins (RBPs)

TAR DNA-binding protein 43 (TDP-43): TDP-43 is a highly conserved and widely expressed heterogeneous ribonucleoprotein (hnRNP), which contains two RNA recognition motifs (RRMS) and a low complexity domain (LCD) rich in C-terminal glycine [50,51]. It was originally identified as a transcription inhibitor [52] and splicing regulator [53], and later considered to be the main component of protein inclusions in the cytoplasm of motor neurons in ALS patients. TDP-43 is the first to be marked as an ALS-related RBP [54,55]. TDP-43 is normally expressed in many tissues including neurons and glial cell nuclei [56]. Most ALS patients are found to have TDP-43 deposits in neuronal inclusion bodies, indicating that TDP-43 exert a pivotal role in ALS pathology [57]. TDP-43 presents with abnormal phosphorylation, ubiquitination, lysis, and/or nuclear depletion in neurons and glial cells, which are prominent pathological features of ALS [58]. In ALS, a functional loss of TDP-43 has been discovered, which may damage RNA metabolism. This loss is also related to the cytoplasmic accumulation of protein aggregates that cause neurotoxicity [59]. In addition, the cytoplasmic positioning error and accumulation of TDP43 destroys self-regulation in ALS. On the contrary, the inefficient self-regulation of TDP43 can lead to cytoplasmic TDP43 deposition and subsequently cause neurodegeneration [60]. However, it is still unclear about the extent to which TDP-43 phosphorylation, ubiquitination, loss of function is involved in the progression of ALS.

Fused in sarcoma (FUS): FUS encodes an RNA binding protein containing 526 amino acids, which belongs to the FET family. FUS is mainly located in the nucleus under normal physiological conditions [61]. FUS, which was initially identified as a fusion protein, is caused by chromosomal translocation in human mucosal liposarcoma in which the N-terminal part of FUS is translocated and fused with a transcription factor CHOP [62]. Studies have shown that FUS protein aggregates are common in patients with ALS, and the conversion from liquid to solid is accelerated by inducing mutations in prion-like regions in the early stage of ALS or by increasing protein concentration. This abnormal phase transition may be the core of ALS [63]. Deficiency of FUS can cause neuronal cell death in Drosophila and zebrafish [64,65]. In addition, an RNA analysis on FUS mutations in motor neurons from induced pluripotent stem cells in ALS indicates that abnormal gene expression and splicing changes are related to FUS mutations [66,67]. This suggests that the abnormal phase transition and RNA metabolism of FUS protein may be involved in the pathogenesis of ALS.

TATA-box binding protein associated factor 15 (TAF15): TAF15 is composed of n-terminal glutamine, glycine, serine and tyrosine (QGSY) enriched domains, a glycine (gly) enriched domain, an RNA recognition motif (RRM), a zinc finger motif (Znf), a characteristic arginine/glycine (RGG) repeat domain, and a nuclear localization signal (NLS) [68,69,70]. Studies have found that TAF15 is mutated in patients with sALS and fALS [71,72]. TAF15 accumulation in the body of Drosophila conferred neurodegeneration in Drosophila, with the ALS-linked variants having a more severe effect than wild type. Immunohistochemical detection of postmortem spinal cord tissue reveals the mislocation of TAF15 in motor neurons of ALS patients [71]. TAF15 and FUS exhibit similar RNA interactions in the mouse brain, and the interaction between them and their RNA binding sites can occur independently of cofactor binding [73]. Undoubtedly, there is a connection between TAF15, FUS, and RNA in ALS, whereas how their combination acts on ALS has not been explored as TAF15 itself has a complex structure.

Ewing Sarcoma breakpoint region 1/EWS RNA binding protein 1 (EWSR1): EWSR1 assembles with FUS and TAF15 to constitute the FET protein family, and its binding to RNA helps control transcription and RNA processing [74]. Studies have shown that the EWSR1 protein presents a diffuse distribution or dotted granule structure in the motor neuron cytoplasm of patients with sALS, and wild-type EWSR1 has inherent pathogenic properties [75]. In Drosophila, overexpression of wild-type EWSR1 leads to neurodegeneration; however, overexpression of mutant EWSR1 does not aggravate the phenotype [76]. EWSR1 seems to have similar pathogenic characteristics to FUS and TAF15, which is easy to aggregate and be toxic. More importantly, they belong to the same family, and their similar domains further support this speculation.

Ataxin-2 (ATXN2): ATXN2 is widely expressed and localized in the endoplasmic reticulum and Golgi cells, and functions via a variety of cellular pathways [77]. ATXN2 plays diversified roles in regulating RNA metabolism, including mRNA stability, polyadenylation, and translation activation [78,79,80,81]. ALS has been proved to be involved in the CAG repeat expansion of the cytoplasmic content of ATXN2-positive neurons [82]. The ATXN2 trinucleotide repeat amplification in ALS is found unable to predict the age of onset, but can predict the risk of the disease [83]. ATXN2 is abnormally located in the spinal cord neurons of ALS patients, and the expansion of ATXN2 intermediate-length polyglutamine increases the risk of ALS [84]. In addition, ATXN2 is also an effective dose-sensitive modifier of TDP-43 toxicity that is across multiple model systems [84]. The interaction between ATXN2 and TDP-43 may bring us a new breakthrough in the treatment of ALS.

Heterogeneous nuclear ribonucleoproteins (HnRNPs): hnRNPA1 and hnRNPA2/B1 interact with the C-terminus of TDP-43, which is mediated through their prion-like domains (PrLD) [85]. Missense mutations in PrLDs produce effective sterically hindered zippers, enhancing the natural tendency to form template fibrils, and promoting the recruitment of stress granules and the formation of cytoplasmic inclusion bodies. Discrete missense mutations alter the gated aspartic acid residues in the PrLD of hnRNPA2/B1 and hnRNPA1, triggering multiple system proteinopathies and ALS [86]. For ALS patients, the hnRNPA1 immunoreactivity is reduced in motor neuron nuclei containing bone-like inclusions, but not detected in bone-like inclusions. The significant loss of hnRNPA1 in motor neurons with the cytoplasmic accumulation of TDP-43 may be a serious obstacle to mRNA processing, indicating that it plays a vital role in the progressive neuronal death of ALS [85]. Studies have shown that hnRNPA3 is clearly mislocalized, and there is no difference in hnRNPA1 or A2/B1 localization. In Australian patients with ALS, no new or known mutations in hnRNPA1, hnRNPA2/B1, or hnRNPA3 are detected [87]. hnRNPs are therefore relatively rare in ALS. However, hnRNPs are definitely involved in the pathogenesis of ALS to some extent, perhaps through the combination with other common pathogenic genes (such as TDP-43, C9orf72), which needs to be explored in further animal experiments.

Matrin-3 (MATR3): MATR3 is a 125 kDA nuclear matrix protein, which contains two zinc finger domains and two RNA recognition motifs (RRM), binding and stabilizing RNA in many tissues [88,89,90]. MATR3 itself does not have a virus-like domain; however, it can interact with TDP-43 in several internally disordered domains. MATR3 is found to partially mis-localize in the motor neuron cytoplasm and surrounding glial cells in ALS patients, with no cytoplasmic content [91]. The S85C missense mutation in the MATR3 gene is a genetic cause for ALS. MATR3 S85C knock-in mice reproduce the behavioral and neuropathological features of early ALS, including dyskinesia, muscle atrophy, neuromuscular defects, Purkinje cell degeneration, and neuroinflammation of the cerebellum and the spinal cord [92]. Extremely different from other RBPs, MATR3 has no virus-like domain, and its potential pathogenic mechanism may provide a new direction for the study of ALS.

T-cell-restricted intracellular antigen 1/TIA1-related (TIA1/TIAR): T-cell intracellular antigen 1 (TIA1) and TIA1-related protein (TIAR) were originally confirmed to be two components of cytotoxic T lymphocyte particles. TIA1/TIAR is important stress granule (SG) components [93], and TIA1 plays a central role through its LCD in promoting SG assembly [94,95,96]. TIAR may be involved in neuronal cell death after ischemia [97]. In a new type of ALS/FTD family, the P362L mutation in the LCD of TIA1 has been identified. Subsequent genetic association analysis reveals that there is an increased risk for TIA1 LCD mutations in ALS patients [98]. Oxidative stress is definitely involved in the pathogenicity of TIA1/TIAR, and the two can be linked for research in the future.

### 3.2. ALS-Related Genes

Superoxide dismutase 1 (SOD1): SOD1 is a powerful antioxidant enzyme that protects cells against the damage of superoxide free radicals [99]. At least 170 mutations in the SOD1 gene have been found to cause ALS. When mutated, the abnormal SOD1 enzyme acquires new harmful properties. The 155 of the 170 confirmed mutations are known mutations, most of which are found in familial cases, although about 3% are from sporadic cases [100]. The toxicity of mutant SOD1 may be aroused by the initial misfolding that reduces the nuclear protection from active enzymes (loss of nuclear function). This process may be related to the pathogenesis of ALS [101]. Interestingly, misfolded SOD1 protein in fALS can spread between molecules in a prion-like manner [102]. In addition, ER-Golgi transport dysfunction observed in the embryonic cortex and motor neurons of SOD1^G93A^ mice maybe indicates the early stage of ALS (which may be the main cause) [103]. Studies have found that from the aspect of mitochondrial dynamics, the dysfunction mediated by mutant SOD1^G93A^ in ALS is related to enhanced apoptosis in osteocytes, which may be a potential mechanism of bone loss during the progression of ALS [104]. To date, it is still unclear how SOD1 mutation selectively causes motor neuron death, which is also a key point in the pathogenesis of SOD1-ALS.

C9orf72: The C9orf72 complex is involved in various cellular processes, including vesicle transport, lysosomal homeostasis, mTORC1 signaling transduction, and autophagy. C9orf72 deficiency can lead to excessive inflammation in mice, while patients with C9orf72 mutation have a neuroinflammatory phenotype [105]. In 2011, an abnormal GGGGCC hexanucleotide repeat expansion in C9orf72 was identified as the most common genetic cause of fALS [106,107]. A study showed that in the Drosophila G4 C2 repeated amplification model, DPR protein but not RNA lesions was the main toxic molecule, and defects in nuclear and cytoplasmic transport were found in neurons derived from C9orf72-iPSC [108]. To understand how this repetitive RNA and its translation products (various DPR proteins) cause neurodegeneration may help us identify potential therapeutic targets for C9orf72-ALS.

Coiled-coil-helix-coiled-coil-helix domain containing 10 (CHCHD10): CHCHD10 is a mitochondrial protein located in the inter-membrane space, which was first found to be associated with ALS in a family of French origin [109]. CHCHD10 acts as a new gene related to the clinical lineage of ALS-FTD, and brings a potential mitochondrial basis for such diseases. In sporadic and familial cases, a CHCHD10 analysis is also required in patients with ALS or FTD [109]. However, CHCHD10 mutations appear to be the relatively rare cause of ALS and may be more common in patients diagnosed with frontotemporal dementia [110,111]. Next challenge for ALS may be to combine CHCHD10 with mitochondrial function.

TANK-binding kinase 1 (TBK1): TBK1 is a member of the inhibitor of nuclear factor-κB (IκB) kinase (IKK) family and participates in the innate immune signal pathway [112]. A mutation in TBK1 is the main genetic cause of ALS/FTD comorbidities (10.8%), while is less associated with ALS alone (0.5%) [113,114]. Studies have shown that the genetic susceptibility resulting from TBK1 heterozygosity can be exacerbated by the decrease of TGFβ-activated kinase 1 (TAK1) expression in the aging-induced brain, thereby promoting receptor-interacting protein kinase 1 (RIPK1) and activating ALS/FTD [115]. Regardless of the type of mutation, insufficient haplodose of TBK1 is the cause of ALS and FTD [114]. Pathological analyses of the brain and spinal cord from ALS patients with TBK1 mutations also reveal TDP-43 positive inclusion bodies [116,117]. Mutations in TBK1 may be related to immune disorders and also involved in phosphorylation. Therefore, TBK1 seems to be another gene related to TDP-43.

Tubulin alpha-4A (TUBA4A): TUBA4A is a gene encoding tubulin alpha-4A protein [118]. The mutations in TUBA4A are associated with fALS. All patients with TUBA4A mutations experience spinal seizures accompanied by upper and lower motor neuron signs (classical ALS) [118]. Downregulation of microRNA-1825 is detected in the central nervous system and extra-central system of patients with sALS and fALS, leading to the upregulation of tubulin folding cofactor b (TBCB). Excessive TBCB then causes depolymerization and degradation of TUBA4A [119]. TUBA4A may influence the pathogenesis of ALS by the involvement in the metabolism of microRNA, which requires more experiments to verify.

NIMA-related kinase 1 (NEK1): NEK1 is a member of the highly conserved never in mitosis A (NIMA) kinase family composed of 11 genetically distinct proteins. It has a conserved function in cell cycle progression and mitosis. Study has determined a significant association between NEK1 variants with loss of function (LOF) and fALS risk [120]. Nine NEK1 variants have been detected in Chinese patients with ALS, including three new heterozygous loss-of-function mutations and six rare missense variants in NEK1 [121]. However, few animal experiments related to NEK1 have been done, and we need to find a way to establish an animal model of this gene for in-depth studies in further.

Chromosome 21 open reading frame 2 gene (C21orf2): A recent study has determined that C21orf2 is associated with ALS [122]. C21orf2 interacts with NEK1, participating in microtubule assembly, DNA damage response and repair, and mitochondrial function [123,124]. Based on bioinformatics and molecular modeling approaches, over 75% of the mutations are found to be potentially detrimental [125]. C21orf2 is similar to C9orf72 and NEK1 in protein structure and function, and therefore, we reasonably infer that their pathogenic mechanisms may be interrelated.

Cyclin F (CCNF): CCNF is a gene encoding cyclin F, a component of the E3 ubiquitin-protein ligase complex (SCF). Expression of mutant CCNF in neuronal cells induces abnormal ubiquitination and accumulation of ubiquitinated proteins, including TDP-43 and SCF [126]. Mutations in CCNF may increase the ATPase activity of valine-containing proteins (VCP) in the cytoplasm, thereby increasing TDP-43 aggregates and eventually causing the onset of ALS [126]. In Western and Japanese populations, CCNF mutations are associated with ALS. However, mutations in CCNF are rarely reported in patients with ALS in China’s mainland [127]. CCNF variant is considered to be a rare cause of ALS, with varying variant rates in populations from different regions.

Kinesin family member 5A (KIF5A): KIF5A is a member of the kinesin family. A recent whole-genome analysis has determined that KIF5A is a new gene associated with ALS, and a mutation in the C-terminal cargo binding tail domain of KIF5A leads to ALS [128]. A whole exon sequence analysis of several cases of fALS patients indicates an enrichment of *KIF5A* splice-site mutations in ALS [129]. A novel KIF5A loss-of-function variant has been discovered recently, and the loss-of-function variant in KIF5A is reported to be a rare cause of sALS in Japanese patients [130]. Misfolded wild-type SOD1 positive cytoplasmic inclusions are found in motor neurons of patients with no SOD1 mutations but carrying KIF5A pathogenic mutations [131]. As a novel ALS-associated gene, KIF5A’s pathogenic mechanism for ALS is still unclear. The connection between KIF5A and SOD1 is worth exploring.

Annexin A11 (ANXA11): ANXA11 is a phosphoinositide binding protein associated with RNA particles, which is a molecular chain between RNA particles and lysosomes. ALS-associated mutations in ANXA11 can disrupt the interaction between RNA transport and lysosome, thereby impairing RNA transport [132]. Expression of ANXA11 variants in motor neuron cells gives rise to the cytoplasmic separation of endogenous FUS and triggers neuronal apoptosis. Mutations in ANXA11 can be involved in the pathogenesis of ALS through a gain-of-function mechanism involving abnormal protein aggregation [133]. ANXA11 can block RNA transport, which may be one of the important mechanisms underlying the pathogenesis of ANXA11.

Glycosyltransferase 8 domain containing 1 (GLT8D1): GLT8D1 encodes a glycosyltransferase with unknown function. ALS-associated GLT8D1 variants are toxic in vivo and in vitro. Mutations in the GLT8D1 glycosyltransferase domain are related to fALS [134]. In a cohort of European descent, genetic variants of GLT8D1 and ARPP21 (cAMP regulated phosphoprotein 21) are found to be associated with ALS. However, mutations in GLT8D1 and ARPP21 are not found in fALS and sALS populations of Australian origin [135]. No pathogenic mutations in GLT8D1 and ARPP21 are found in Chinese patients with sALS [136]. Therefore, the activity of glycosyltransferase is considered to be associated with the development of ALS, especially fALS. In addition, the mutation of GLT8D1 will also vary in different regions.

SPG11 (spatacsin): SPG11 is positioned on chromosome 15q13-15 and encodes the spatacsin protein, exerting a key role in axon maintenance, synaptic vesicle transport and autophagy [137]. Mutations in SPG11 are considered to be the pathogenic factor of spastic paraplegia characterized by autosomal recessive inheritance and juvenile ALS [138]. In addition to recessive inheritance and early onset, adolescent ALS is usually different from adult ALS because of its slow progression rate and long course of the disease that can last for decades [139]. At present, relatively few studies have been reported on ALS-associated SPG11 mutations. This may be an important therapeutic target for adolescents.

### 3.3. Non-Coding RNA

MicroRNAs (miRNAs): miRNAs serve as the regulators of gene expression at the post-transcriptional level, which are essential for neuronal differentiation, survival, and activity [140]. MiR-27a, miR-34a, miR-155, miR-142-5p, and miR-338-3p have been studied as biomarkers and potential therapeutic targets related to ALS [141,142,143,144]. Studies have shown that miR-27a-3p displayed downregulation in serum exosomes of ALS patients, suggesting exosomal miR-27a-3p may be a detection indicator of ALS. However, the specific mechanism is still unclear, and further explorations are encouraged [145]. A recent study indicated that miR-124 was down-regulated in neural stem cells and up-regulated in differentiated neural stem cells in SOD1^G93A^ mice in vitro. At the same time, the protein levels of Sox2 and Sox9 and the expression of miR-124 changed in the reverse direction in vivo and in vitro. Studies have shown that Sox2 and Sox9 regulate the differentiation of neural stem cells into astrocytes during the development of ALS. MiR-124 is involved in the selective differentiation of neurons and astrocytes. Therefore, miR-124 plays an important role in the differentiation of astrocytes in ALS transgenic mice by targeting Sox2 and Sox9 [146]. MiR181a-5p, which is up-regulated in the cerebrospinal fluid of ALS patients, is proposed as an anticancer drug, serving as a tumor suppressor in normal tissues, and promoting growth inhibition and apoptosis [147]. Another study focused on the changes in the neuroprotective neuroinflammation in the spinal cord of SOD1^G93A^ mice during the pre-symptomatic and symptomatic phases, and found that both astrocytes and microglia were changed in the pre-symptomatic phase in which the up-regulated miR-155 is expected to be a marker of early ALS [148]. The dysregulation of miRNA expression in ALS patients can provide important insights into the pathogenesis of the disease and ultimately contribute to the development of potential therapies in the future.

lncRNAs: A previous study found that a lncRNA recruited FUS/TLS (for translocated in liposarcoma) to the genomic locus encoding cyclin D1, where cyclin D1 transcription was inhibited in response to DNA damage signals, leading to an increased tolerance to cell apoptosis, which suggests that the destruction of FUS/TLS-induced single-stranded lncRNA may play a role in the pathological changes of neurodegenerative diseases [149,150]. TDP-43 nuclear bodies (NBs) are found to be partially collinear with nuclear paraspeckles, whose scaffold, lncRNA/nuclear-enriched abundant transcript 1 (NEAT1), is significantly upregulated in stressed neurons. These findings indicate a stress-mitigating role and mechanism of TDP-43 NBs, and the dysfunction of TDP-43 NBs is likely to be involved in the pathogenesis of ALS [151]. NEAT1_2 (nuclear-enriched abundant transcript 1_2) can be used as a scaffold for RNA and RNA-binding proteins in the nucleus of ALS motor neurons, thereby regulating the function of ALS-associated RNA-binding proteins in the early stage of ALS [152]. ATXN2-AS is the antisense gene of ATXN2, and the expanded ATXN2-AS (expATXN2-AS) is expressed in lymphoblasts of ALS patients, triggering the toxicity [153]. In patients with mutations in the FUS gene, there are 21 lncRNAs identified, 11 of which are antisense. Seven antisense lncRNAs have been detected in patients with TDP-43 mutations, and only one has been described as SNAP25-AS, i.e., antisense lncRNA of synaptic protein SNAP25 [154]. In ALS patients with SOD1 mutations, two new antisense lncRNAs have been found, one of which is identified as a CKMT2 antisense RNA, a mitochondrial creatine kinase [155]. These findings may enlighten the development of new therapeutic targets for the treatment of ALS.

Circular RNA (circRNAs): circRNA is a unique type of non-coding regulatory RNA known to be particularly abundant in the nervous system, which is characterized by a structure with covalently closed ends [156]. Cytoplasmic accumulation of TDP-43 is discovered in some forms of sALS [157]. DBR1 deficiency serves as the strongest inhibitor of TDP-43 toxicity [158]. FUS is determined to be a new regulator of circRNAs production and plays an important role in controlling the expression of these molecules in mouse motor neurons, which may lead to a new insight into the mechanism of FUS-ALS [159]. A recent study showed that hsa_circ_0023919, hsa_circ_0088036 and hsa_circ_0063411 are potential blood-based biomarkers of ALS [160]. Compared with linear RNA, the unique circular structure can protect circRNA from ribonuclease digestion and provide a longer half-life [161]. Although there are no many clear circRNAs as biomarkers of ALS, further explorations on its unique structure and potential association with ALS-associated genes are encouraged.

### 3.4. Others

Serum uric acid (UA): There is a negative correlation between serum UA levels and the risk of death in patients with ALS [162]. The serum UA level of ALS patients is lower than normal. This decrease is related to disease progression, further confirming the possible role of oxidative stress in the induction and spread of the disease [163].

Cardiolipin (CL): CL is a mitochondria-exclusive phospholipid. A recent lipidomics analysis has shown that, in addition to other lipidomics changes, there is a significant reduction in CL levels in the spinal cord of rats with ALS symptoms [164]. Alterations in CL levels may also reflect the loss of mitochondrial integrity observed in several ALS models [165]. Abnormal CL metabolism plays a broad role in the pathogenesis of ALS.

Chitotriosidase (CHIT1): An elevated level of CHIT1 in the cerebrospinal fluid (CSF) of ALS patients has been indicated, and CHIT1 level in CSF may exert an additional effect in the prognosis of ALS patients with a short history of symptoms that are difficult to identify [166]. The level of CHIT1 in the CSF of patients with ALS may reflect the degree of activation of microglia/macrophages in the white matter of the spinal cord. CHIT1 may be a potentially useful marker for the differential diagnosis of ALS and for the prediction of disease progression [167].

Neurofilament light chain (NfL): Blood NfL can be used as a biomarker with prognostic value for ALS. In patients with ALS, serum NFL is positively correlated with disease progression, while a higher NFL level indicates a shorter survival period [168]. The blood NfL level of ALS patients is about four times that of the control group, and maintains a relatively stable expression during follow-up, which provides the potential for NfL as a biomarker of drug efficacy in future trials [169]. This conclusion has also been verified in Chinese patients with ALS using ultra-sensitive Simoa technology [170].

**Table 1:** Biomarkers of ALS.

Biomarkers		Description	References
RBPs	TDP-43	TDP-43 is the main component of cytoplasmic protein inclusions in ALS patients; abnormal phosphorylation, ubiquitination, lysis, and/or nuclear depletion are prominent pathological features of ALS.	[54,55,58]
	FUS	FUS protein aggregates are common in ALS patients; the abnormal phase transition and RNA metabolism of FUS protein may be involved in the pathogenesis of ALS.	[63,66,67]
	TAF15	TAF15 is mutated in patients with sALS and fALS; TAF15 accumulation conferred neurodegeneration.	[71,72,73]
	EWSR1	EWSR1 protein presents a diffuse distribution or dotted granule structure of patients with sALS; overexpression of wild-type EWSR1 leads to neurodegeneration.	[75]
	ATXN2	The expansion of ATXN2 intermediate-length polyglutamine increases the risk of ALS; ATXN2 trinucleotide repeat amplification in ALS can predict the risk of the disease.	[83,84]
	HnRNPs	HnRNPs is relatively rare in ALS, but definitely involved in the pathogenesis of ALS, perhaps through the combination with other common pathogenic genes (TDP-43).	[85,171]
	MATR3	MATR3 is found to partially mis-localize in ALS patients; S85C missense mutation in the MATR3 gene is a genetic cause for ALS.	[91,92]
	TIA1/ TIAR	TIA1/TIAR are important stress granule components; TIAR may be involved in neuronal cell death after ischemia, while an increased risk for TIA1 LCD mutations was found in ALS patients.	[93,97,98]
ALS-related genes	SOD1	SOD1 is a powerful antioxidant enzyme; At least 170 mutations in the SOD1 gene have been found to cause ALS; the toxicity of mutant SOD1, which may be aroused by the initial misfolding, are related to the pathogenesis of ALS.	[99,101]
	C9orf72	An abnormal GGGGCC hexanucleotide repeat expansion in C9orf72 was identified as the most common genetic cause of fALS.	[106,107]
	CHCHD10	CHCHD10 is a mitochondrial protein located in the inter-membrane space; CHCHD10 mutations appear to be the relatively rare cause of ALS, and may be more common in patients diagnosed with frontotemporal dementia.	[109,110,111]
	TBK1	TBK1 is a member of the inhibitor of nuclear factor-κB kinase family; a mutation in TBK1 is the main genetic cause of ALS/FTD comorbidities (10.8%), while is less associated with ALS alone (0.5%).	[113,114]
	TUBA4A	TUBA4A is a gene encoding tubulin Alpha 4A protein; mutations in TUBA4A are associated with fALS, and all patients with TUBA4A mutations experience spinal seizures accompanied by upper and lower motor neuron signs.	[118]
	NEK1	A significant association has been determined between NEK1 variants with loss of function and fALS risk.	[120].
	C21orf2	C21orf2 is associated with ALS; Over 75% of the mutations are found to be potentially detrimental.	[122,125]
	CCNF	CCNF is a gene encoding cyclin F, a component of the E3 ubiquitin-protein ligase complex. Mutations in CCNF may increase TDP-43 aggregates and cause the onset of ALS; CCNF variant is considered to be a rare cause of ALS, with varying variant rates in populations from different regions.	[126]
	KIF5A	KIF5A is a member of the kinesin family; KIF5A is a new gene associated with ALS; a mutation in the C-terminal cargo binding tail domain of KIF5A leads to ALS.	[128]
	ANXA11	ANXA11 is a phosphoinositide binding protein associated with RNA particles; mutations in ANXA11 can be involved in the pathogenesis of ALS through a gain-of-function mechanism involving abnormal protein aggregation.	[133]
	GLT8D1	GLT8D1 encodes a glycosyltransferase, the activity of glycosyltransferase is considered to be associated with the development of ALS, especially fALS.	[134]
	SPG11	SPG11 encodes the spatacsin protein; mutations in SPG11 are considered to be the pathogenic factor of spastic paraplegia characterized by autosomal recessive inheritance and juvenile ALS	[138]
Non-coding RNA	miRNAs	MiR-27a, miR-34a, miR-124, miR-142-5p, miR-155 and miR-338-3p have been studied as biomarkers and potential therapeutic targets related to ALS.	[141,142,143,144,146,148]
	lncRNAs	NEAT1_2 can regulate the function of ALS-associated RNA-binding proteins in the early stage of ALS; In ALS patients with FUS, TDP-43, and SOD1 mutations, 20 antisense lncRNAs have been found in total.	[152,154,155]
	circRNAs	CircRNAs biogenesis that is regulated by inhibiting the function of DBR1 is considered to be a potential therapeutic strategy for ALS; hsa_circ_0023919, hsa_circ_0088036 and hsa_circ_0063411 are potential blood-based biomarkers of ALS.	[158,160]
Others	UA	There is a negative correlation between serum UA levels and the risk of death in patients with ALS.	[162]
	CL	Alterations in CL levels may also reflect the loss of mitochondrial integrity observed in several ALS models.	[165]
	CHIT1	An elevated level of CHIT1 in the cerebrospinal fluid of ALS patients has been indicated.	[167]
	NfL	Serum NFL is positively correlated with disease progression, while a higher NFL level indicates a shorter survival period.	[168]

## 4. Progress of Treatments

In the past few decades, a breakthrough has been made in understanding the pathogenesis and biomarkers of ALS. However, therapeutic strategies are still limited. Therefore, it is an urgent task for us to find an effective treatment for ALS. In recent years, novel targets and strategies that are developed based on the research progress in the genetics, pathology, and molecular mechanism of ALS have been used as emerging ALS therapeutic interventions. Here, we review the current status and mechanisms of some ALS treatments that appeared in preclinical or early clinical development, including drug therapy, gene therapy, immunotherapy, and stem cell-exosomal therapy (Table 2).

### 4.1. Drug Treatments

Riluzole and Edaravone are approved by the Food and Drug Administration (FDA) for the treatment of ALS; however, both show a variety of side effects. Riluzole is the only drug to extend the survival of patients with ALS. Clinical trials have shown that riluzole can increase the median survival from 11.8 months to 14.8 months [172]. Edaravone is an antioxidant and free radical scavenger, which can effectively slow the progression of symptoms, weight loss and motor neuron degeneration in SOD1^G93A^ mice, and reduce SOD1 deposition [173]. Spasticity is one of the main challenges in the treatment of ALS. Baclofen, tizanidine, and benzodiazepines can be used to alleviate spasticity in patients with ALS. However, there are considerable limitations in terms of efficacy and tolerability. THC:CBD (trade name Sativex) is more valuable for the treatment of spasticity in patients with ALS [174]. Masitinib is a tyrosine kinase inhibitor. When delivered orally, masitinib can reduce microgliosis and motor neuron pathology and prolong survival after paralysis in SOD1^G93A^ mice [175]. Fasudil is a potent rho kinase (ROCK) inhibitor that slows disease progression in SOD1^G93A^ mice, prolongs survival and reduces motor neuron loss. Fasudil also attenuates the increase in ROCK activity and phosphorylated phosphatase and tensin homologue deleted on chromosome 10 (PTEN) and the decrease in Akt in SOD1G93A mice [176]. Further studies will focus on the development of clinical drugs with anti-oxidation and anti-inflammatory properties, targeting the regulation of the immune system and redox disorders.

### 4.2. Gene Therapies

Antisense oligonucleotides (ASOs): A single dose of SOD1 ASO can reverse the initial loss of compound muscle action potentials in SOD1^G93A^ mice. ASOs targeting SOD1 can prolong survival in SOD1 mouse and rat models, and these ASOs are also effective in non-human primates [177]. A single-dose ASO injection that selectively targets RNA containing repetitive sequences can cause rapid reduction of RNA lesions and DPR proteins, while maintaining the overall level of mRNA encoding C9orf72 and continuing to alleviate behavioral defects [178]. Injection of ASOs targeting ATXN2 into the central nervous system of a mouse model with TDP-43 proteinopathy can alleviate TDP-43 pathology and improve motor function [179]. Studies have shown that ASO-based strategies have been successfully used to silence GGGGCC repeat expansion of C9orf72, thereby inhibiting the chelation of RBP by abnormal RNA lesions [180,181]. In addition to directly targeting ALS-associated genes, we can also consider targeting some RNA or proteins that cause ALS.

Clustered regularly interspaced short palindromic repeats/CRISPR-associated system 9 (CRISPR/Cas9) genome editing: CRISPR/Cas9 can delete a copy of Ku80 (DNA repair protein) in C9orf72 iPSCs, leading to the decrease in Ku80 expression and pro-apoptotic protein levels. Thus, CRISPR/Cas9 approach can be used for C9orf72 repeat expansion to verify the pathological phenotype in patient-derived iPSCs and to determine gene-based therapies [182]. Studies have shown that once SOD1 gene is deleted, adeno-associated virus 9-Staphylococcus aureus Cas9-single guide RNA5 (AAV9-SaCas9-sgRNA5) will increase survival of SOD1^G93A^ mice by 54.6%, and significantly improve the performance of ALS transgenic mice. Gene editing based on AAV9-SaCas9-sgRNA therefore demonstrates its potential for treating mutant SOD1 ALS [183]. Existing evidence has proved that base editing can be used to treat a mouse model of ALS. The split-intron method of Streptococcus pyogenes Cas9-cytidine base editors (SpCas9-CBE) delivered by double AAV vector particles is a potential candidate as a gene therapy for other nerves and neurodegenerative diseases [184]. Gene therapy for sexual diseases offers the possibility [184]. Genome editing technology still rises to many challenges, and we need to verify its efficacy through further experiments in vivo and in vitro.

### 4.3. Immunotherapy

In clinical trials, immune disorders lead to abnormal function of regulatory T lymphocytes and increase pro-inflammatory macrophages. Therefore, a better understanding of biological processes that cause these immune disorders will help determine therapeutic strategies to evade or improve the pathogenesis of ALS [185]. Lack of C5aR1 (up-regulated in human and rodent ALS) prolongs the survival of SOD1^G93A^ mice [186]. A significant increase in the level of C5a in the plasma and on the surface of white blood cells (C5aR1 binding) in ALS patients [187] implies that the C5a/C5aR1 axis can be identified as a therapeutic target for ALS. In addition, poly-glycine-alanine (poly-GA) vaccination is confirmed to be safe and effective in the C9orf72 mouse model. Pre-symptomatic administration of poly-GA vaccine can reduce inclusion bodies, and greatly prevent TDP-43 mislocalization, neuroinflammation, nerve axon damage, and motor deficits in GA-CFP mice. Therefore, it is a promising prevention strategy that the vaccine should be vaccinated in the early stage of C9orf72-ALS [188]. We can maintain our focus on passive immunotherapy besides active immunotherapy such as vaccination, attempting to achieve the therapeutic effect by directly injecting purified antibodies into a target organism.

### 4.4. Stem Cell-Exosome Therapy (ASC-Exosome)

Stem cells are a promising treatment for neurogenerative diseases in which adipose-derived stem cells (ASCs) can be obtained in large quantities and are available for autologous cell transplantation. A study comparing two ASC-exosomes administration routes in SOD1G93A mice found that the movement performance of ALS mice was significantly improved and the activation of glial cells was reduced [189]. Expression of mutant SOD1^G93A^ can induce mitochondrial dysfunction, interfere with complex I-mediated oxidative phosphorylation, and reduce coupling efficiency and mitochondrial membrane potential. However, ASC-exosomes can reverse this mitochondrial dysfunction in motor neuron-like cell lines (NSC-34) induced by mutant SOD1^G93A^. These extracellular vesicles represent a promising strategy for the treatment of ALS [190]. A comprehensive proteomics analysis linked the anti-apoptotic effects of ASC-exosomes after internalization in an in vitro model of ALS and clarified how these exosomes regulate their beneficial effects, for the first time providing a theoretical basis for their use in the treatment of neurodegenerative diseases [191]. For fALS, ASC may promote neuroprotection directly and/or by regulating the secretome of local glial cells toward a neuroprotective phenotype [192]. Autologous and undifferentiated ASCs have obvious potential to delay the progression of fALS in animal models [192]. Although stem cell therapy has aroused great expectations, there is still limited information on the molecular mechanisms involved.

**Table 2:** Therapeutic strategies of ALS.

Therapeutic Strategy		Description	References
Drug treatments	Riluzole	The only drug to extend the survival of patients with ALS.	[172]
	Edaravone	An antioxidant which can slow the progression of symptoms.	[173]
	Baclofen, tizanidine, benzodiazepines, THC: CBD	The drugs can alleviate spasticity in patients with ALS.	[174]
	Masitinib	A tyrosine kinase inhibitor can reduce microgliosis, motor neuron pathology, and prolong survival after paralysis.	[175]
	Fasudil	A ROCK inhibitor that slows disease progression and prolongs survival and reduces motor neuron loss.	[176]
Gene therapies	Antisense oligonucleotides (ASOs)	ASOs directly targeting ALS-associated genes can alleviate pathology, improve motor function, and prolong survival.	[177,179]
	CRISPR/Cas9 genome editing	CRISPR/Cas9 approach can be used for C9orf72 repeat expansion to determine gene-based therapies; gene editing based on AAV9-SaCas9-sgRNA demonstrates its potential for treating mutant SOD1 ALS.	[182,183]
Immunotherapy		C5a/C5aR1 axis can be identified as a therapeutic target for ALS; the vaccine should be vaccinated in the early stage of C9orf72-ALS.	[186,187,188]
Stem cell-exosome therapy (ASC-exosome)		ASC-exosomes can reverse the mitochondrial dysfunction, reduce the activation of glial cells, and improve the movement performance in vitro and vivo ALS model.	[189,190]

## 5. Prospects and Challenges

Although numerous studies regarding ALS and its possible pathogeneses have been conducted, the exact mechanism underlying the occurrence and development of the disease is still unclear. ALS is a multi-causal syndrome, indicating that a therapeutic mechanism underlying the successful treatment for one type of ALS may be unavailable for another type. We urgently need to find newer biomarkers and drugs with newer indications in the treatments of ALS. For fALS, the iPSC-based motor neuron model captures the typical disease phenotype; however, iPSC models for sALS still faces great challenges [193]. Many biomarkers and treatments for ALS have been discovered, and current concerns are to affirm their therapeutic effects. In future clinical trials, a good trial design will be the most promising approach to achieve desired outcomes in alleviating incurable diseases.

## 6. Conclusions

The purpose of this article is to summarize the current molecular mechanism of ALS, including immune disorders, redox imbalance, autophagy dysfunction, and disordered iron homeostasis, propose some related biomarkers including RNA binding proteins (RBPs), ALS-related genes, and non-coding RNA, and discuss therapeutic strategies including drug therapy, gene therapy, immunotherapy, and stem cell-exosomal therapy. These therapeutic strategies can more or less slow down the progression of this serious disease. As there is no cure for ALS, this research field is very important for human beings. It is hoped that this review can provide direction for the follow-up research and open up a new research route for the current treatment.
